# Failures and complications associated with resorbable and non-resorbable membranes in guided bone regeneration: A systematic review and meta-analysis

**DOI:** 10.4317/medoral.27721

**Published:** 2026-03-07

**Authors:** Daniela Sánchez Cousiño, Sonia Egido-Moreno, Beatriz González Navarro, Holmes Ortega Mejía, Andrés Blanco-Carrión, José López-López

**Affiliations:** 1 Faculty of Medicine and Health Sciences, University of Barcelona, 08907 Barcelona, Cataluña, Spain; 2 Department of Odontostomatology. Faculty of Medicine and Health Sciences (Dentistry), University of Barcelona 08907 Barcelona, Cataluña, Spain; 3 Oral Medicine, Oral Surgery and Implantology Unit (MedOralRes), Faculty of Medicine and Dentistry Universidade de Santiago de Compostela, Santiago de Compostela, Spain. Instituto de Investigación Sanitaria de Santiago (IDIS), ORALRES Group, Santiago de Compostela, Spain; 4 Dental Hospital of the University of Barcelona (HOUB), Department of Odontostomatology. Faculty of Medicine and Health Sciences (Dentistry), University of Barcelona, 08907 Barcelona, Cataluña, Spain

## Abstract

**Background:**

Patients treated with guided bone regeneration (GBR) may be treated using resorbable or non- resorbable membranes, which differ in their efficacy, failures and complication profiles. The objective is to evaluate the efficacy and failures and complications of resorbable membranes versus non-resorbable membranes in patients undergoing guided bone regeneration (GBR). A search was conducted until July 31, 2025. Randomized clinical trials (RCTs) comparing both types of membrane in ROG were the eligibility criteria.

**Material and Methods:**

Data collection and risk of bias assessment (RoB 2.0) were performed. Primary endpoints included bone regeneration efficacy and membrane-associated complications.

**Results:**

Twenty-five RCTs with a total of 684 patients were included. Most studies reported a high success rate for both types of membranes. However, non-resorbable membranes had a higher incidence of complications, such as membrane exposure (up to 71%), dehiscence and infections.

**Conclusions:**

Both types of membranes are effective for ROG, they should be chosen according to the defect and type of patient. Resorbable membranes have a lower complication rate and non-resorbable membranes offer greater dimensional stability and the need to perform a second surgery for its removal.

## Introduction

Alveolar bone atrophy is a frequent consequence of tooth loss and manifests as progressive bone resorption that can reach moderate or severe degrees. This resorption is due to the absence of the functional stimulus that the teeth exert on the alveolar bone, which triggers a continuous process of bone loss. The speed and pattern of this resorption depends, among other factors, on the anatomical location (1).

To address bone volume loss after extraction, multiple augmentation techniques have been developed, which can be applied to both the maxilla and the mandible, depending on the type and extent of the defect. Among the most used are bone grafts, either in the form of blocks (onlays) or particulate, osteodistraction, guided bone regeneration (GBR), transposition of the inferior alveolar nerve and the use of containment devices such as titanium meshes (1).

In the context of GBR, two main approaches can be used depending on the type of membrane selected: Nonresorbable membranes, such as titanium-reinforced polytetrafluoroethylene (PTFE) membranes, and resorbable membranes, usually collagen. In vertical regeneration procedures, resorbable membranes require additional support to maintain the necessary space for bone formation, such as a titanium mesh or an osteosynthesis plate (1).

However, the use of barrier devices involves a demanding surgical technique and is not without complications. The main cause of failure in GBR procedures is premature or late exposure of the membrane, which can lead to contamination of the biomaterial and irreversibly compromise bone regeneration (1).

Furthermore, in the case of non-resorbable membranes, a second surgical intervention is required for their removal, usually six to eight months after placement (2). In contrast, resorbable membranes have the advantage of eliminating the need for additional surgery, and present a lower risk of exposure, perforation and infection. However, the choice of membrane type depends on multiple clinical factors, including the size of the defect and the presence or absence of supporting bone walls. For this reason, both non-resorbable membranes and titanium meshes continue to be therapeutic options especially indicated in extensive bone defects or in atrophic ridges lacking walls, although regeneration in these cases tends to be slower (3).

## Material and Methods

The review was prepared according to Cochrane Collaboration guidelines and reported following the PRISMA extension statement for reporting systematic reviews incorporating network meta-analyses of health care interventions (PRISMA-NMA) (4).

The following question was developed: In patients undergoing guided bone regeneration, are resorbable membranes more effective and have fewer failures and complications than non-resorbable membranes?

Study eligibility criteria (in PICO format)

P (Population): Patients undergoing guided bone regeneration (GBR) procedures with the use of membranes. I (Intervention): Use of resorbable membranes for guided bone regeneration. C (Comparison): Use of non-resorbable membranes for guided bone regeneration. O (Outcome - Results). Efficacy: success rate of bone regeneration (e.g., new bone formation, graft stability). Failures: Membrane exposure, graft loss, infections. Clinical complications: Inflammation, undesirable bone resorption, need for reoperation.

Inclusion and exclusion criteria

Inclusion criteria: Population: Patients undergoing guided bone regeneration (GBR) using membranes. Intervention and comparison: Use of resorbable membranes vs. non-resorbable membranes in guided bone regeneration procedures or membranes vs. a control group. Results: Reports results related to: Efficacy: New bone formation, graft stability. Failures: Membrane exposure, infections, graft loss, unwanted bone resorption. iv.-Study design: Randomized controlled trials (RCTs). Clinical studies with comparisons between membrane or control groups. v.-Language: Published in English or Spanish.

Exclusion criteria: Does not compare resorbable and non-resorbable membranes directly or membranes with respect to a control group. Does not report specific results on bone regeneration (efficacy or failures). Animal or in vitro studies. Descriptive studies, systematic reviews, case series or studies without a comparative group.

Search strategy and information resources

Electronic searches were conducted until July 31, 2025 in four databases: CENTRAL (Cochrane Central Register of Controlled Trials), OVID MEDLINE, EMBASE and Web of Science. For PubMed, MeSH terms and free terms combined using Boolean operators were used, with the following search strategy: (("Guided Bone Regeneration"[MeSH] OR "Alveolar Ridge Augmentation"[MeSH] OR "Bone Regeneration") AND ("Absorbable Implants"[MeSH] OR "Biodegradable Materials"[MeSH] OR "bioabsorbable membranes" OR "resorbable membranes") AND ("Non-Absorbable Membranes" OR "Polytetrafluoroethylene"[MeSH] OR "non-bioabsorbable membranes" OR "non-resorbable membranes")) AND ("Bone Formation"[MeSH] OR "Bone Regeneration"[MeSH] OR "Treatment Outcome"[MeSH] OR "Postoperative Complications"[MeSH] OR "Treatment Failure"[MeSH]) AND (Humans[MeSH]) AND (english[Language] OR spanish[Language]). For the Cochrane database, the same search strategy was used: (("Guided Bone Regeneration"[MeSH] OR "Alveolar Ridge Augmentation"[MeSH] OR "Bone Regeneration") AND ("Absorbable Implants"[MeSH] OR "Biodegradable Materials"[MeSH] OR "bioabsorbable membranes" OR "resorbable membranes") AND ("Non-Absorbable Membranes" OR "Polytetrafluoroethylene"[MeSH] OR "non-bioabsorbable membranes" OR "non-resorbable membranes")) AND ("Bone Formation"[MeSH] OR "Bone Regeneration"[MeSH] OR "Treatment Outcome"[MeSH] OR "Postoperative Complications"[MeSH] OR "Treatment Failure"[MeSH]).

Study selection and data extraction

The selection of titles and abstracts was performed by a reviewer (DSC). In case of doubt, the decision on which articles to review in full text was made by discussion with the review team (SEM). A reviewer (DSC) then reviewed the full text of the articles for inclusion. Again, in case of uncertainty, the decision on inclusion or exclusion was made by discussion with the review team. Data extraction forms were then developed, tested on several articles, and modified if necessary, before use. Next, one reviewer (DSC) extracted the data, and two other reviewers (SEM, JLL) checked a randomly selected sample (50%) of the extracted data. The following data were extracted: Context, study design, population characteristics (no. of patients, types of defects, location), details of interventions, and information on primary and secondary outcomes.

Quantitative methods

Pooled analyses were performed using a random-effects model. Study heterogeneity was assessed using the I2 statistic. Heterogeneity between studies was considered statistically significant for a pvalue&lt;0.05 and was interpreted according to Cochrane Handbook recommendations: 0%-40% was considered unimportant, 30%-60% as moderate heterogeneity, 50%-90% as substantial heterogeneity, and 75%-100% as considerable heterogeneity. Review Manager 5.4 was used as a tool to analyze the data, previously recorded an Excel table. Forest Plots were made to graphically represent the difference between the different types of membranes, with a 95% confidence interval (CI).

Assessment of risk of bias and certainty of evidence

Risk of bias was assessed at the outcome level in all included RCTs using the Cochrane Risk of Bias (RoB) 2.0 tool (5). Certainty in evidence (CiE) was assessed at the outcome level using the Grading of Recommendations, Assessment, Development and Evaluation (GRADE) approach (6). Assessment of publication bias using funnel plots was not performed because the number of studies included in each meta-analysis was limited, which precluded a meaningful evaluation.

## Results

Study selection

**Figure 1:**
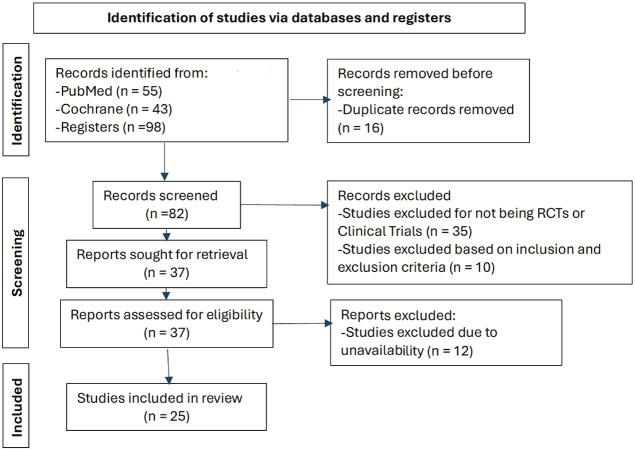
Flow Chart diagram.

A total of 98 records were identified, of which all were electronically searched. After eliminating duplicates, the titles and/or abstracts of 82 records were examined. Once irrelevant titles and abstracts were excluded, the full texts of 37 publications were examined, allowing us to exclude 12 articles for which the full text was not available and to include 25 publications describing 25 randomized controlled trials (RCTs) (Figure 1) (7). Twenty-five RCTs contributed to the quantitative analyses. In addition, 14 articles not included in the analyses were obtained from manual searches to contrast results from other authors.

[caption id="attachment_2016" align="alignnone" width="300"]1 Characteristics of the studies included[/caption]Twenty-five randomized clinical trials (RCTs), published between 1995 and 2025 were included, with a total of 684 patients evaluated. In Table 1 we present characteristics of the 25 studies included in the review (1 - 3 , 8 - 29).

Table 1

The sample size per study ranged from 9 to 72 participants, the largest study being that of Jung et al. (19), while the smallest was by Majzoub et al. (24). Follow-up time ranged from 4 to 168 months, although most studies established short- or medium-term follow-up (12 months).

The most common anatomic locations were infraosseous and furcation class II defects, followed by postextraction and peri-implant defects. Most of the procedures were performed in the posterior mandibular region and in the anterior maxilla.

Regarding the type of intervention, the studies mainly compared non-resorbable membranes (especially e-PTFE and d-PTFE) with resorbable membranes (collagen, polyglactin 910, polylactide/polyglycolide). In addition, some studies evaluated variants with titanium mesh or textile membranes. Of all the studies, 19 used bone grafts (autogenous, xenografts, allogeneic or combinations), while 6 did not use any additional biomaterial.

In relation to bone formation, the results were variable. For example, Cucchi et al. (3) reported a vertical gain of 7.8±1.9mm with Ti-PTFE membranes, while 7.4±2.3mm was obtained with collagen and titanium mesh. Silvestri et al. (13) recorded a gain of 4.8±2.1mm with e-PTFE. In contrast, Van der Zee et al. (9) observed a volume loss of −0.34mm, which highlights the influence of the type of membrane, the defect treated and the surgical protocol.

Regarding complications, these were frequent with non-resorbable membranes. Membrane exposure was the most reported, present in at least 12 studies, with rates reaching up to 71% in the study by Friedmann et al. and 70% in the study by Silvestri et al. (13). Other common complications included dehiscence, infection and gingival recession. In contrast, studies such as those by Mandarino et al. (20) Vaibhav et al. (21) and Merli et al. (27) reported no relevant adverse events.

Most studies reported success rates above 90%, especially in the groups treated with resorbable membranes. However, the definition of "success" was variable among studies, including clinical, radiographic or histologic criteria.

Risk of bias in the studies and certainty of evidence

Of the 25 studies included in the review, 11 were at low risk of bias, whereas 6 presented moderate risk and 8 were classified as high risk (Table 2).

Table 2

The main sources of bias were associated with methodological limitations related to lack of blinding, small sample sizes, and lack of long-term follow-up. These shortcomings compromise the internal validity of the studies and, consequently, influence the overall certainty of the evidence.

The quality of the evidence was degraded mainly by these limitations in the design and by the imprecision of the results.

Quantitative data synthesis

A meta-analysis was performed with the studies that provided comparable data between membranes. The following parameters were studied: Bone formation, success rate and complications.

**Figure 2:**
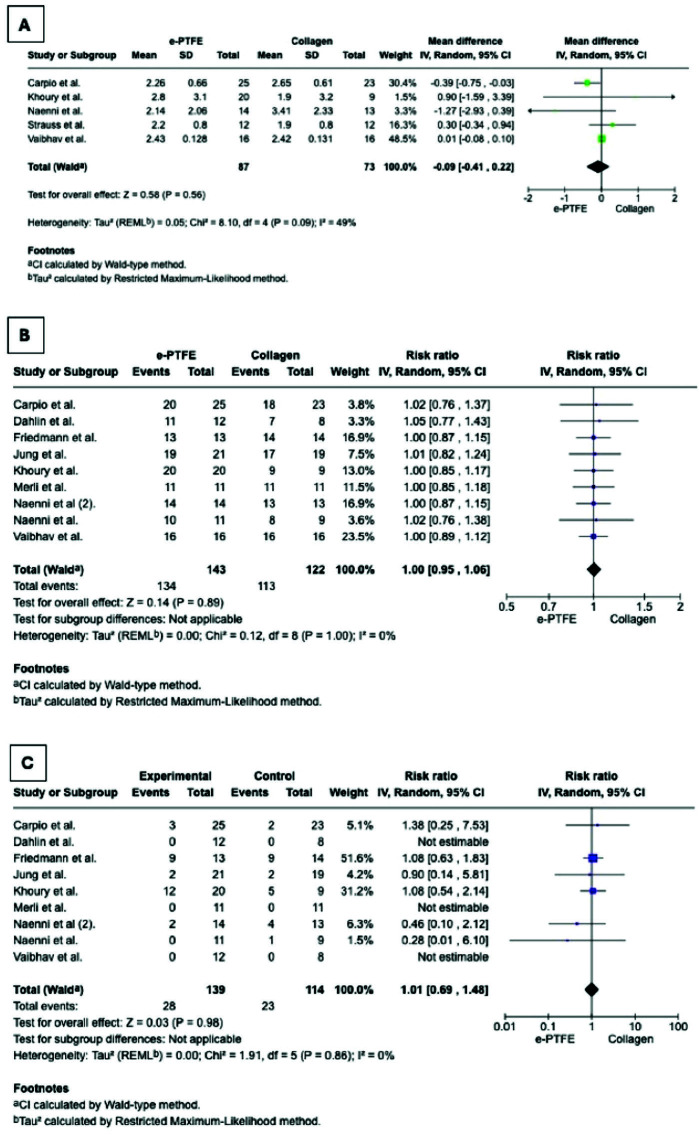
(A) Meta-analysis of bone formation comparing e-PTFE membranes versus collagen membranes. (B) Meta-analysis of success rate comparing e-PTFE membranes versus collagen membranes. (C) Meta-analysis of complications comparing e-PTFE membranes versus collagen membranes.

In the comparison between e-PTFE and collagen membranes, no statistically significant differences were observed in terms of Bone formation: The mean difference was -0.09mm; with a 95% confidence interval (CI): -0.41 to 0.22; P=0.56, although the heterogeneity between studies was high (I2=49%) (Figure 2A).

2In the success rate: The risk ratio was 1.00 with a 95% CI of 0.95 to 1.06; P=0.89, with zero heterogeneity between studies (I2=0%) (Figure 2B). And the complications: The "risk ratio" was 1.01 with 95% CI: 0.69 to 1.48; P=0.98, with null heterogeneity between studies (I2=0%) (Figure 2C).

**Figure 3:**
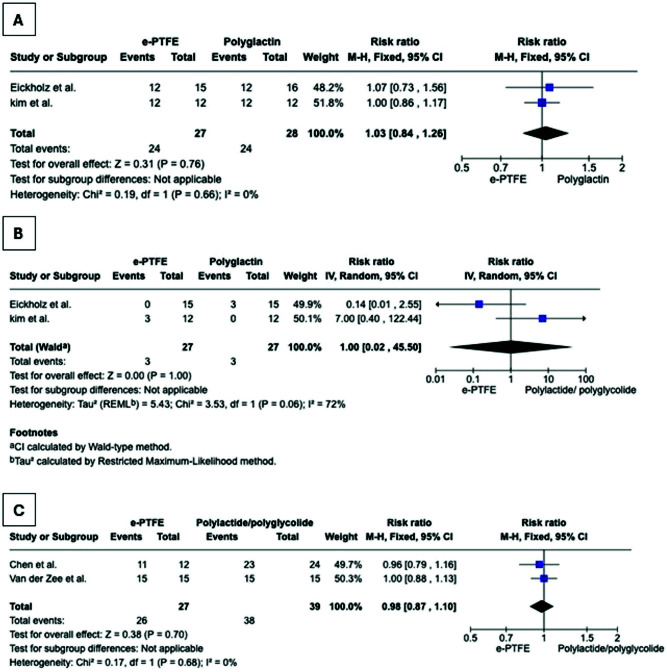
(A) Meta-analysis of success rate comparing e-PTFE membranes versus polyglactin 910 membranes. (B) Meta-analysis of complications comparing e-PTFE membranes versus polyglactin 910 membranes. (C) Meta-analysis of success rate comparing e-PTFE membranes versus polylactide/polyglactin 910 membranes.

When comparing e-PTFE with polyglactin 910 membranes, no statistically significant differences were found: Success rate: The risk ratio was 1.03 with a 95% CI of 0.84 to 1.26; P=0.76, with no heterogeneity between studies (I2=0%) (Figure 3A). And the complications: The "risk ratio" was 1.00 with 95% CI: 0.02 to 45.50; P=1.00, although heterogeneity among studies was high (I2=72%) (Figure 3B). And the the comparison of e-PTFE with polylactide/polyglactin 910 no statistically significant differences were found: Success rate: The risk ratio was 0.98 with a 95% CI of 0.87 to 1.10; P=0.70, with zero heterogeneity between studies (I2=0%) (Figure 3C).

3

## Discussion

This systematic review compared the clinical efficacy and complications associated with the use of resorbable and non-resorbable membranes in guided bone regeneration (GBR) procedures. Overall, both showed satisfactory results in bone formation and graft stability, although with different complication profiles.

Clinical efficacy: Bone formation and graft stability

Regarding treatment efficacy, evaluated by bone formation and graft stability, no significant differences were found between resorbable and non-resorbable membranes in studies with follow-up of less than 24 months (2). However, the quality of regenerated bone and its long-term stability remain critical aspects for success (3).

Studies with Ti-PTFE membranes or titanium meshes showed similar proportions of new bone, residual biomaterial and soft tissue. For example, with a 50:50 mixture of autologous bone and allograft, 39.7% new bone was obtained with Ti-PTFE and 42.1% with collagen membrane and titanium mesh (3). Urban et al. (30) reported similar results: 36.6% new bone, 16.6% residual material and 46.8% soft tissue when Ti-PTFE was used with autogenous bone and DBBM (3).

The optimal healing time continues to be debated, but an average of 9 months is recommended; 6 months is acceptable for horizontal and 9 months for vertical augmentations. Some studies have even reported good results in vertical augmentations after 6 months (3). In the study by Cucchi et al. (3), the titanium mesh group showed more bone tissue and less soft tissue than the Ti-PTFE group, possibly due to better revascularization associated with resorbable membranes, as opposed to the greater rigidity of PTFE.

Regarding long-term stability, Kim et al. (11) observed that vertical gain with polyglactin 910 remained relatively stable, with a mean loss of only 1.0mm between 6 and 60 months. Similar results were obtained with ePTFE membranes: Losses of 0.9mm between 12 and 24 months (17), and 1.2mm after ROG versus 1.3mm after conventional surgery (18). These losses were mainly related to smoking or low adherence to maintenance (11).

Kim et al. (11) also studied the IL-1 polymorphism, linked to an increased inflammatory response. In the study by Sanctis et al. (31), 75% of patients were genotype-positive, compared with the expected 33%. Only two negative patients showed significant loss, which reinforces the role of this polymorphism as a risk factor.

In simultaneous ROG and implant installation procedures, Naenni et al. (17) documented a significant reduction of the bone defect (2.14-3.41mm) from initial surgery to reentry. Although both groups lost horizontal thickness, it was less in the group without resorbable membrane (N-RES), suggesting that collagen membranes may collapse even with bone support. At 5 years, bone changes were minimal, but more stable in the N-RES group (-0.24mm vs. -0.28mm in RES), possibly because of the greater barrier capacity of ePTFE (18).

In the long term, Jung et al. (19) reported a survival rate of 93% at 12.5 years, higher than that of other studies reporting 89.3% at 10 years and 82.9% at 16 years, demonstrating the potential for ROG stability over time.

-Complications according to membrane type

The choice of membrane type implies different complication profiles. Non-resorbable membranes, such as ePTFE and titanium meshes, have a higher rate of exposure, which may compromise bone regeneration (1). These exposures, both early and late, are the main cause of procedure failure and can lead to infections, abscesses or total graft loss, even without visible exposure (1)

Exposure rates range from 0-45% for PTFE membranes and 12-50% for titanium meshes, according to Strauss et al. Simion et al. and Roccuzzo et al. (29 , 32 , 33). In early exposures (first 4 weeks), a direct correlation was observed between the exposed area and bone loss, with a deficit of 16.3% per cm² exposed. Paresthesias were also reported (one with d-PTFE, three with Ti mesh), linked to closures under tension or surgical difficulty (1).

Collagen membranes had more implant exposures, although less dehiscence than ePTFE during the first six months. Similar results were obtained by Zitzmann et al. (34) Fixation with polylactic acid pins improved healing (63.6% vs. 28.6% in unfixed membranes), reducing postoperative micromovement (9).

Another disadvantage of non-resorbable membranes is the need for a second surgery for their removal, which can induce gingival recession and flap necrosis. In addition, they require intensive surveillance in case of exposure or suppuration, since spontaneous healing is limited (23 , 24). Strietzel (35) reported exposure rates of up to 50% with ePTFE membranes. In contrast, biodegradable membranes favored a more predictable healing without the need for removal for Simion et al. (36).

In combination with enamel matrix derivatives (DBBM), collagen membranes achieved defect resolution rates of 85-96%, higher than those observed with non-resorbable membranes, which had greater exposure and less bone fill (10 , 37). Despite a higher number of dehiscences with collagen (30% vs. 14%), thickness loss was lower in the N-RES group, indicating greater dimensional stability. The collapsibility of resorbable membranes remains a limitation, even with support (10).

Although ePTFE membranes have proven clinical success, their vulnerability to bacterial colonization due to their macroporosity compromises outcomes (10). Survival rates with these membranes vary between 79.4% and 100%, but they present greater bone loss associated with soft tissue complications. In contrast, collagen membranes achieved a rate of 95.4% in the study by Zitzmann et al. (38).

After the date our search was completed, a new trial was published by Hindryckx et al. (39) comparing dPTFE and collagen membranes in lateral bone augmentations in the maxillary anterior region. Although both groups showed similar results in terms of bone gain at 9 months, the group treated with dPTFE had a higher rate of infections (33%, P=0.019), which reinforces the evidence on the complications associated with the use of non-resorbable membranes.

In immediate implants, exposures were observed in areas with connective tissue grafts (9.7%), with abscesses in 3.2% and reduction of bone volume in exposed areas. Only one out of four areas showed unsatisfactory regeneration, suggesting that resorbable membranes might offer better protection against bacterial migration in case of exposure (23).

Finally, although with small samples, the data indicate that smokers have a worse response to regenerative therapy, regardless of the type of membrane. Tonetti et al. (40) observed lower long-term clinical gains in smokers with ePTFE membranes, despite similar bone regeneration.

The findings of the meta-analysis indicate that there are no statistically significant differences between collagen and e-PTFE membranes in terms of bone formation (mean difference: -0.09mm; 95% CI: -0.41 to 0.22; P=0.56), success rate (RR=1.00; 95% CI: 0.95 to 1.06; P=0.89) and complications (RR=1.01; 95% CI: 0.69 to 1.48; P=0.98), with null heterogeneity for the last two parameters (I2=0%).

Similarly, when comparing e-PTFE with polyglactin 910, no statistically significant differences were found in the success rate (RR=1.03; 95% CI: 0.84 to 1.26; P=0.76; I2=0%) or in the occurrence of complications (RR=1.00 with 95% CI: 0.02 to 45.50; P=1.00), although it showed high heterogeneity (I2=72%), suggesting some variability between studies. Finally, in the comparison between e-PTFE and polylactide/polyglycolide, no significant differences were found in the success rate (RR=0.98; 95% CI: 0.87 to 1.10; P=0.70; I2=0%).

An important limitation of this review is the methodological heterogeneity among the included studies, both in sample size and in the type and location of the bone defects treated. Similarly, the diversity in the biomaterials used, the follow-up time and the variability in the surgical techniques employed make direct comparison of the results difficult. The scarcity of clinical trials with long-term follow-up also represents a disadvantage in establishing definitive conclusions on the stability of the results over time. In addition, study selection was primarily conducted by a single reviewer, with consultation in cases of uncertainty, which may have introduced a risk of selection bias. Sensitivity and subgroup analyses were limited by the heterogeneity of study designs, membrane types, outcome measures, and the relatively small number of comparable studies available form quantitative synthesis. Nevertheless, the certainty of evidence was assessed at the outcome level using the GRADE approach, providing a structured evaluation of the confidence in the estimated effects.

Future research should focus on randomized clinical trials with a larger sample size and long-term follow-up to compare the efficacy and complications associated with the different types of membranes. It would also be opportune to delve into the impact of systemic and genetic factors, such as IL-1 polymorphism on the success of bone regeneration.

## Conclusions

Both resorbable and non-resorbable membranes are effective and achieve predictable bone regeneration, with high success rates in most studies. Since there are no statistically significant differences, the choice of membrane type should be individualized according to the defect, the type of intervention and the patient's clinical history. This is due to the differences in the complication profile, especially the higher frequency of exposures and infections with non-resorbable membranes, even though they offer greater dimensional stability and the need for a second surgery for their removal. In contrast, resorbable membranes have a lower rate of complications.

## Figures and Tables

**Table 1:** Characteristics of the 25 studies included in the review. T of S: Type of study. RCT: Randomized clinical trial. d-PTFE: Dense polytetrafluoroethylene. e-PTFE: Expanded polytetrafluoroethylene. Ti mesh: Titanium mesh. PBL-V: Vertical infraosseous defect. MGCSH: Medical grade calcium sulfate hemihydrate.

Study (author, year)	Type of membrane	T of S. [N]	Localization	Follow-up (months)	Bone graft	Success rate (%)	Complications (%)	Bone formation (mm)	Risk of bias
Cucchi et al. 2017 (1)	d-PTFE vs. titanium mesh covered with Cross-linked collagen	RCT [1]	Atrophic posterior mandible	12 months	50% autogenous and 50% allograft	d-PTFE: 100% Ti mesh: 100%	d-PTFE: 5% surgical-15% complications vs. Ti mesh: 15.28% surgical-21.1% complications	d-PTFE: 4.2±1mm vs. Timesh: 4.1±1mm	Low
Eickholz et al. 2007 (2)	e-PTFE vs. Poliglactin 910, polylactide-tributylcitrate	RCT [8]	Infrabony defect, maxilla and mandible	60±3 months	No	94%	e-PTFE: 0% vs. Polidioxanone: 2%	PBL-V gain: 1.78±2.67mm Density: +0.16±0.33mm	Moderate
Van der Zee et al. 2004 (9)	e-PTFE vs. Polylactide/polyglycolide	RCT [9]	Maxilla bone defect	12 months	Xenograft, blood cloth only	100%	25% recession	−0.34mm	Moderate
Cucchi et al. 2019 (3)	Ti-PTFE vs. Collagen plus Ti-mesh	RCT [3]	Posterior mandible	9 months	50% autogenous, 50% allograft	100%	7.5% Ti-PTFE vs. 7.5% Collagen plus Ti-mesh	B.Pi. Ti-PTFE: 7.8±1.9mm vs. Collagen plus Ti-mesh: 7.4±2.3mm	Low
Cucchi et al. 2019 (10)	d-PTFE vs. Collagen Ti-mesh	RCT [10]	Posterior mandible	9 months	50% autogenous, 50% allograft	100%	7.5%	d-PTFE: 4.2±1mm vs. Ti mesh: 4.1±1mm	Low
Kim et al. 2002 (11)	e-PTFE vs. Polyglactin 910	RCT [11]	Infrabony defect	60±3 months	No	100%	e-PTFE: 25%	e-PTFE: 1.5mm vs. Polyglactin 910: 2.1mm	High
Eickholz et al. 2006 (8)	e-PTFE vs. Polyglactin 910	RCT [2]	Class II furcation	120±6 months	No	89%	e-PTFE: 11% vs. Polyglactin 910: 11%	e-PTFE: 1.1±1.3mm vs. Polyglactin 910: 1.7±1.4mm	High
Carpio et al. 2000 (12)	e-PTFE vs Collagen	RCT [12]	Osseous defects surrounding dental implants	6 months	Xenograft 50% Autograft 50%	ePTFE: 83.3%, Collagen: 78.3%	e-PTFE: dehiscence 4.1% and exposure 12.5%Collagen: 8.7% exposure	ePTFE: 2.26±0.66mm vs. Collagen: 2.65±0.61mm	Moderate
Silvestri et al. 2000 (13)	e-PTFE vs. enamel matrix derivative vs. Widman modified flap	RCT [13]	Infrabony defect	12 months	No	100%	e-PTFE: 70% exposure	e-PTFE: 4.8±2.1mm vs. EMD: 4.5±1.6mm vs. WMF: 1.2±1.0mm	Moderate
Couri et al. 2002 (14)	e-PTFE vs. barrier of medical grade calcium sulfate hemihydrate [MGCSH]	RCT [14]	Class II furcation	12 months	Demineralized freeze- dried bone allograft [DFDBA	100%	e-PTFE: 15% infection	ePTFE: 2.54±1.56mm vs. MGCSH: 1.38±1.12mm (horizontal fill)	Low
Friedmann et al. 2003 (15)	e-PTFE vs Collagen	RCT [15]	Infrabony defect	7 months	Xenograft	100%	e-PTFE: 71% vs. Collagen: 64% exposure	e-PTFE: 39%±15% vs. Collagen: 42%±18%	Low
Khoury et al. 2001 (16)	e-PTFE vs. Collagen	RCT [16]	Osseous defects surrounding dental implants	36 months	Autogenous	100%	e-PTFE: 60% dehiscence, fistula, exposure, sequestration vs. Collagen: 55.6%	e-PTFE: 2.8±3.1mm vs. Collagen: 1.9±3.2mm	High
Naenni et al. 2017 (17)	e-PTFE vs. Collagen	RCT [17]	Bucofenestrated defect	6 months	Xenograft	100%	e-PTFE: 14% dehiscence vs. Collagen: 30% dehiscence	e-PTFE: 2.14±2.06mm vs. Collagen: 3.41±2.33mm	Moderate
Naenni et al. 2021 (18)	e-PTFE vs. Collagen	RCT [18]	Dehiscence or fenestration defect	60 months	Xenograft	95%	Collagen: 4%	e-PTFE: -1.08mm Collagen: -1.6mm	Moderate
Jung et al. 2013 (19)	e-PTFE vs. Collagen	RCT [19]	Infrabony defect	144-168 months	Xenograft	e-PTFE: 92.6% Collagen: 91.9%	e-PTFE>Collagen	e-PTFE: 2.4mm vs. Collagen: 3.36mm	Low
Mandarino et al. 2018 (20)	d-PTFE vs. cloth	RCT [20]	Extraction sockets	4 months	No	100%	0%	d-PTFE: 4.30±1.20mm Cloth: 2.50±2.20mm	High
Vaibhav et al. 2021 (21)	e-PTFE vs. Collagen	RCT [21]	Sites for implant placement	24 months	Autogenous	100%	0%	e-PTFE: 2.43±0.128mm vs. Collagen: 2.42±0.131mm	Low
Luongo et al. 2022 (22)	d-PTFE vs. cloth	RCT [22]	Post-extraction alveolar bone defects	30 months	Xenograft	100%	0%	d-PTFE: 16.81%±9.61 % vs. Cloth: 35.16%±12.36%	High
Chen et al. 2004 (23)	e-PTFE vs. Polylactide/ polyglycolide vs. Cloth vs. Bone graft	RCT [23]	Maxillary Extraction sockets	6 months	Autogenous	96.8%	9.7% infection, abscess, exposure	e-PTFE: 1.8±0.2mm vs. Polylactide/ polyglycolide: 1.8±0.3mm vs. Bone graft: 1.6±0.2mm vs. Cloth: 1.5±0.3	Low
Mazjoub et al. 1999 (24)	e-PTFE	RCT [24]	Infrabony defect	8 months	No	100%	Control: 22% Fenestration, infection	e-PTFE: 3.1±1.1mm	High
Dahlin et al. 2010 (25)	e-PTFE vs. Collagen	RCT [25]	Infrabony defect anterior maxilla	6 months	Xenograft 80% autogenous 20%	97.5%	5% exposition	MBL: -3.51 to -2.38mm	Low
Antoun et al. 2001 (26)	e-PTFE vs. Autogenous graft	RCT [26]	Infrabony defect	6 months	Autogenous	100%	e-PTFE: 8.3% exposition	e-PTFE: 3.7mm vs. Graft: 2.9mm	High
Merli et al. 2014 (27)	e-PTFE vs. Collagen	RCT [27]	Infrabony defect	72 months	Autogenous	100%	0%	e-PTFE: -0.49mm vs. Collagen: -0.58mm	Low
Scott et al. 1997 (28)	e-PTFE vs. Laminar bone	RCT [28]	Class II furcation	6 months	Allograft	100%	e-PTFE: significant exposure (up to 1.8mm) weeks 3-5	e-PTFE Vertical: 1.0−1.2mm vs. Horizontal: 2.0−2.2mm	High
Strauss et al. 2025 (29)	e-PTFE vs. Collagen	RCT	Anterior region	6 months	Demineralized bovine bone mineral (DBBM)	e-PTFE: 84.6% Collagen: 66.7%	e-PTFE: 15.4% dehiscence vs. Collagen: 33.3% dehiscence	e-PTFE: -0.1mm (p=0.017) vs. Collagen: -0.8mm	Low

1

**Table 2:** Risk of bias in the articles included in the systematic review. +: Low. Un: Unclear. -: High.

Study	Random Sequence Generation	Allocation Concealment	Blinding of Participants and Researchers	Incomplete Outcome Data	Selective Reporting	Other Bias	General Risk of Bias
Cucchi et al. 2017 (1)	+	+	+	+	+	+	+
Eickholz et al. 2007 (2)	-	Un	Un	-	Un	Un	Un
Van der Zee et al. 2004 (9)	+	Un	Un	-	Un	Un	Un
Cucchi et al. 2019 (3)	Un	+	+	+	Un	+	+
Cucchi et al. 2019 (10)	+	+	Un	+	Un	Un	+
Kim et al. 2002 (11)	-	Un	Un	-	Un	-	-
Eickholz et al. 2006 (8)	Un	-	Un	-	Un	Un	-
Carpio et al. 2000 (12)	Un	+	+	Un	-	Un	Un
Silvestri et al. 2000 (13)	+	Un	-	+	+	Un	Un
Couri et al. 2002 (14)	+	+	+	Un	+	Un	+
Friedmann et al. 2003 (15)	Un	+	Un	+	Un	+	+
Khoury et al. 2001 (16)	-	Un	Un	-	Un	Un	-
Naenni et al. 2017 (17)	Un	+	Un	+	+	Un	Un
Naenni et al. 2021 (18)	Un	+	Un	+	+	Un	Un
Jung et al. 2013 (19)	+	+	Un	+	+	Un	+
Mandarino et al. 2018 (20)	Un	Un	Un	-	Un	Un	-
Vaibhav et al. 2021 (21)	+	+	Un	+	+	+	+
Luongo et al. 2022 (22)	Un	-	+	+	Un	Un	-
Chen et al. 2004 (23)	+	+	+	+	+	+	+
Mazjoub et al. 1999 (24)	-	Un	Un	-	Un	Un	-
Dahlin et al. 2010 (25)	+	+	Un	+	+	+	+
Antoun et al. 2001 (26)	Un	-	Un	-	Un	Un	-
Merli et al. 2014 (27)	+	+	+	+	+	+	+
Scott et al. 1997 (28)	-	Un	Un	-	Un	Un	-
Strauss et al. 2025 (29)	Un	+	Un	+	+	+	+

2

## Data Availability

All relevant data are provided within the article.
